# Polyostotic Fibrous Dysplasia of the Middle Finger

**DOI:** 10.5334/jbsr.4110

**Published:** 2025-10-16

**Authors:** Tristán Igual-Pacheco, Nicolas De Vos, Jonas De Melio

**Affiliations:** 1 Ghent University, 9000 Gent, Belgium; 2 Radiologists at Hospital ‘AZ Sint-Blasius Dendermonde’, 9200 Dendermonde, Belgium

**Keywords:** fibrous dysplasia, polyostotic, bone disease, developmental, phalanges of finger, hand, diagnostic imaging, radiography, magnetic resonance imaging, bone lesion, tomography, X-ray computed, ground-glass appearance

## Abstract

*Teaching point:* When a polyostotic lesion in the fingers is seen in a pediatric patient, fibrous dysplasia should be included in the differential diagnosis and a CT scan should be considered for the assessment of ground-glass densities.

## Case

**Figure 1:**
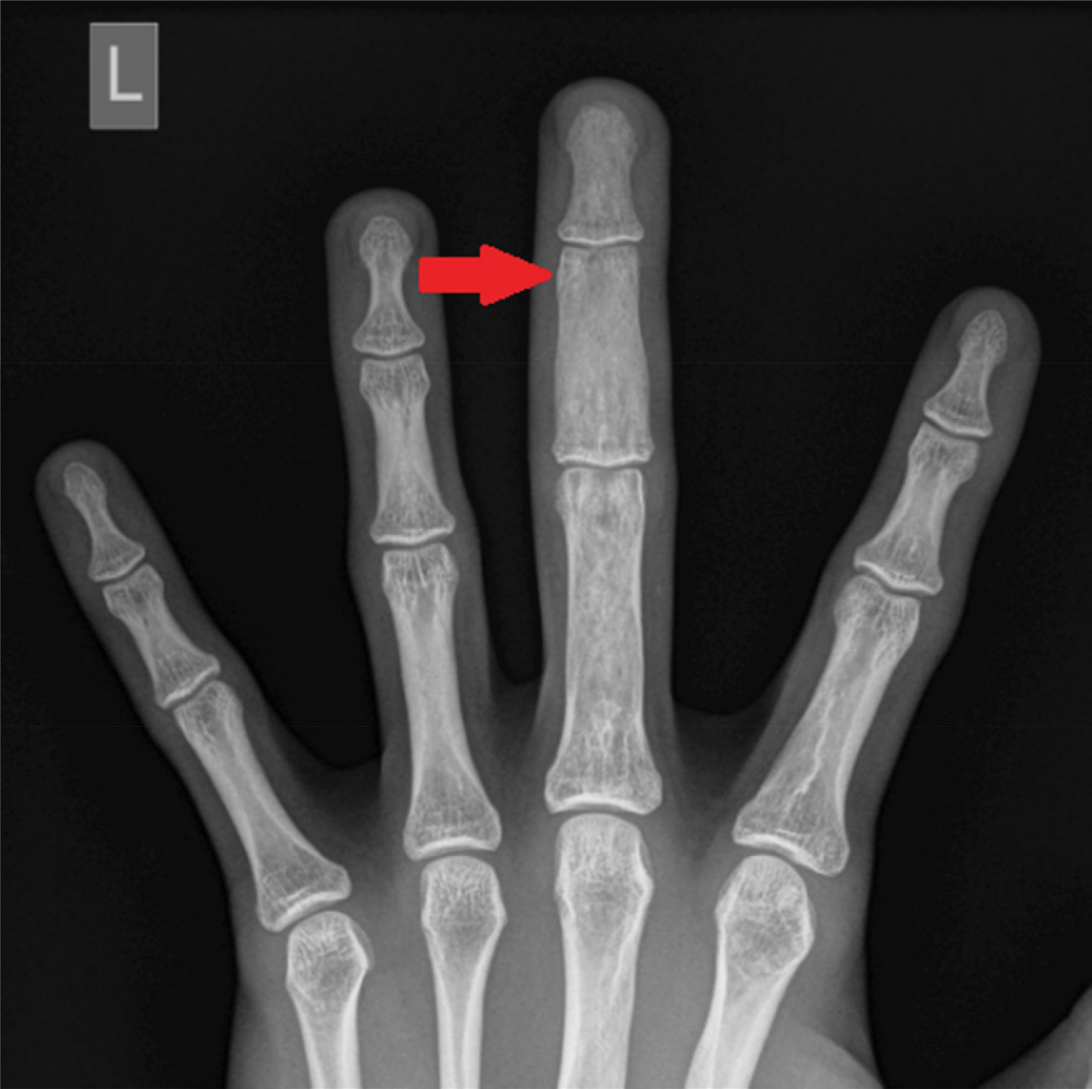
Anteroposterior X-ray of the left hand showing an expansile lesion involving all phalanges of the middle finger and a visible fracture line in the middle phalanx.

**Figure 2 A–C:**
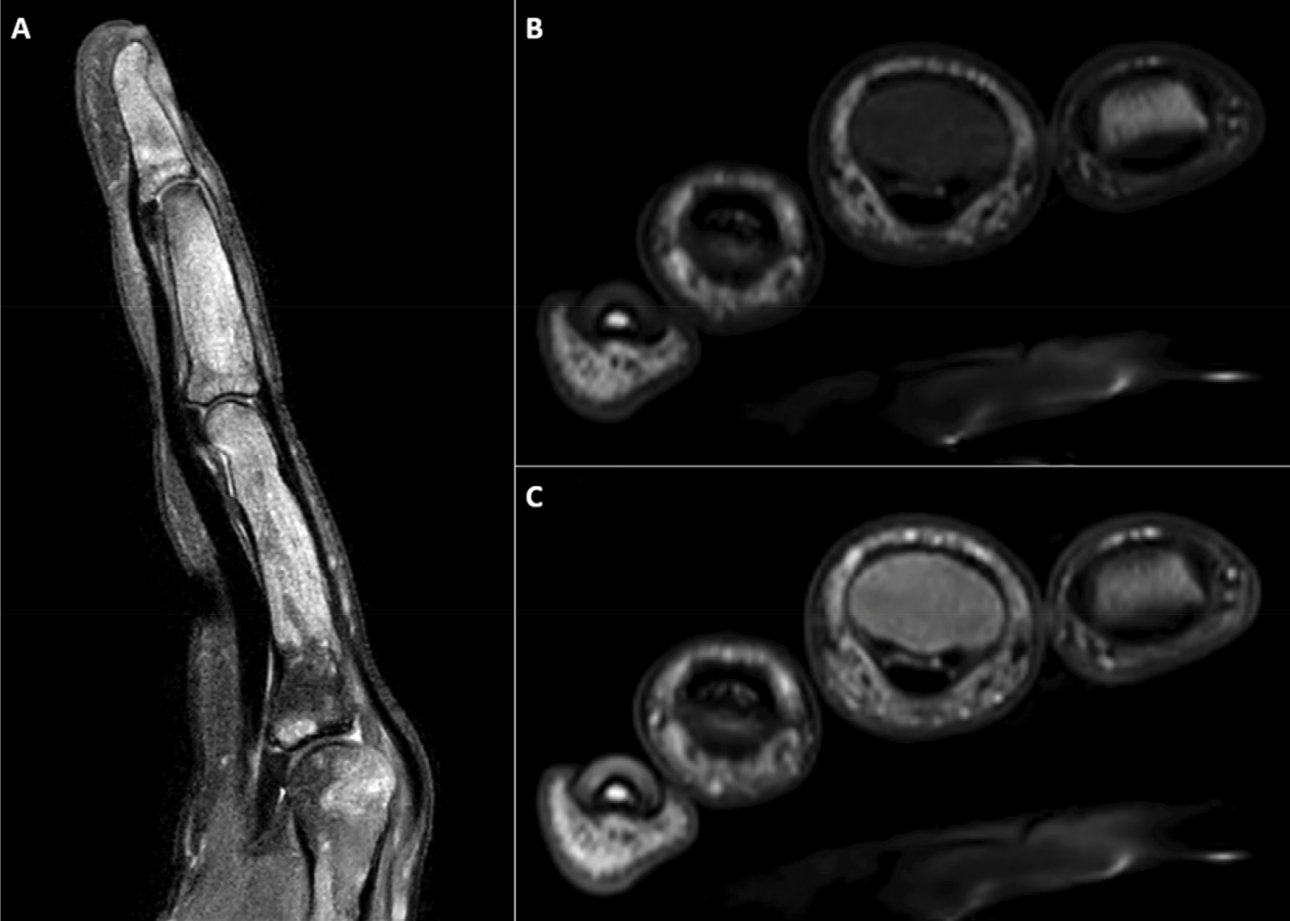
MRI of the left hand demonstrating multiple T2-hyperintense **(A)**, T1-hypointense **(B)**, and enhancing **(C)** lesions involving the phalanges and metacarpal head, leading to trabecular expansion and cortical thinning.

**Figure 3:**
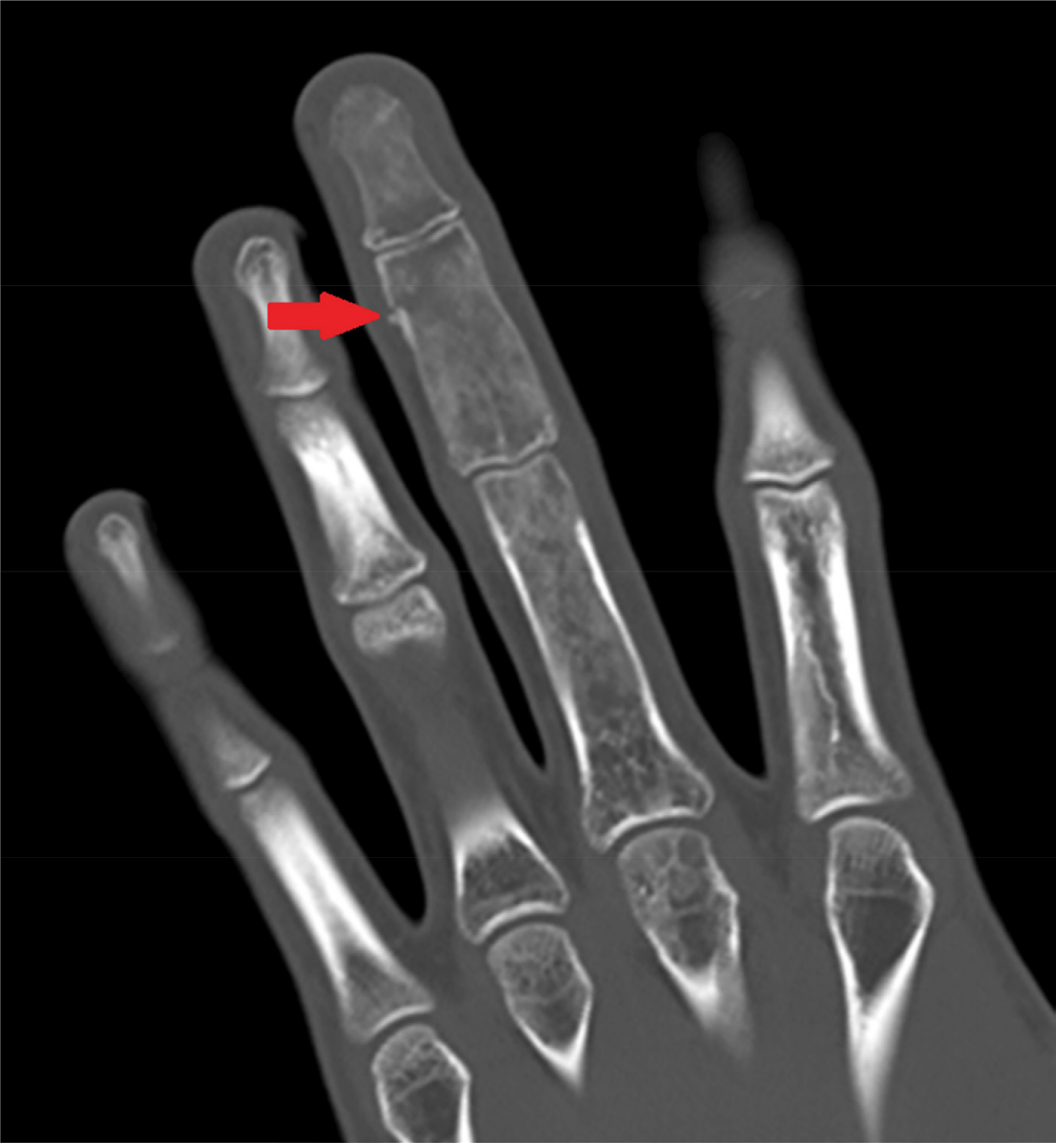
CT scan of the left hand showing multiple ground-glass bone lesions and a cortical fracture of the middle phalanx.

A 16-year-old male patient presented to the emergency department after injuring his left middle finger while playing basketball. X-ray showed a fracture of the middle phalanx and an aberrant expansile aspect of all phalanges of the middle finger (Figure 1). On MRI, multiple T2-hyperintense (Figure 2A), T1-hypointense (Figure 2B) and enhancing lesions (Figure 2C) were seen in the phalanges and metacarpal head, causing an expansion of the trabecular bone and thinning of the cortical bone. On CT, the lesions contained multiple ground-glass densities, and the cortical fracture in the middle phalanx was visible (Figure 3). Given the fact that the patient was known to have a history of a pathological femoral fracture caused by an aneurysmal bone cyst secondary to fibrous dysplasia (FD), the diagnosis of polyostotic FD with a pathologic fracture of the middle phalanx of the middle finger was made.

## Comments

FD is a benign bone disease in which an abnormal differentiation of osteoblasts occurs, leading to the replacement of normal bone marrow and trabecular bone by fibrous stroma and immature bone. FD can either be monostotic (80%) or polyostotic (20%). Usually FD is found incidentally, but complications like a pathologic fracture, aneurysmal bone cysts or malignant degeneration can occur. FD can be one of the characteristics of McCune–Albright syndrome or Mazabraud syndrome.

Monostotic FD (MFD) is most often observed in the ribs, tibia, femur, humerus, mandible and skull. Asymptomatic MFD usually does not cause significant deformity, and most often does not convert to the polyostotic form. MFD usually becomes inactive during puberty.

The polyostotic variant (PFD) is most commonly seen in the skull, facial bones, pelvis and spine. It is often unilateral and is often limited to one limb. PFD is more often associated with fractures and deformations. Similarly to MFD, it usually does not proliferate and becomes inactive around puberty.

According to Fitzpatrick et al. [1], FD appears as intramedullary expensile lesions on medical imaging. A smooth cortical contour is always present, but endosteal scalloping can occur. The lesions appear hazy with ground-glass opacities, but can also be completely radiolucent or sclerotic. On MRI, FD typically shows an intermediate to low intensity on T1-weighted images and intermediate to high intensity on T2-weighted images with heterogeneous enhancement after gadolinium administration.
